# The association of Wnt-signalling and EMT markers with clinical characteristics in women with endometrial cancer

**DOI:** 10.3389/fonc.2023.1013463

**Published:** 2023-03-08

**Authors:** Živa Ledinek, Monika Sobočan, Damjan Sisinger, Marko Hojnik, Tomaž Büdefeld, Uroš Potočnik, Jure Knez

**Affiliations:** ^1^ Department of Pathology, University Medical Centre Maribor, Maribor, Slovenia; ^2^ Divison for Gynaecology and Perinatology, University Medical Centre Maribor, Maribor, Slovenia; ^3^ Department of Gynaecology and Obstetrics, Faculty of Medicine University of Maribor, Maribor, Slovenia; ^4^ Center for Human Molecular Genetics and Pharmacogenomics, Faculty of Medicine, University of Maribor, Maribor, Slovenia; ^5^ Laboratory for Biochemistry, Molecular Biology and Genomics, University of Maribor, Maribor, Slovenia; ^6^ Department for Science and Research, University Medical Centre Maribor, Maribor, Slovenia

**Keywords:** Wnt pathway, EMT - epithelial to mesenchymal transformation, endometrial cancer (EC), β-catenin (B-catenin), DKK1, E-cadherin, N-cadherin

## Abstract

Endometrial cancer is the most common gynecologic malignancy in the developed world. Risk stratification and treatment approaches are changing due to better understanding of tumor biology. Upregulated Wnt signaling plays an important role in cancer initiation and progression with promising potential for development of specific Wnt inhibitor therapy. One of the ways in which Wnt signaling contributes to progression of cancer, is by activating epithelial-to-mesenchymal transition (EMT) in tumor cells, causing the expression of mesenchymal markers, and enabling tumor cells to dissociate and migrate. This study analyzed the expression of Wnt signaling and EMT markers in endometrial cancer. Wnt signaling and EMT markers were significantly correlated with hormone receptors status in EC, but not with other clinico-pathological characteristics. Expression of Wnt antagonist, Dkk1 was significantly different between the ESGO-ESTRO-ESP patient risk assessment categories using integrated molecular risk assessment.

## 1 Introduction

Endometrial cancer (EC) is the most common gynecologic malignancy in the developed world. With average overall 5-year survival rate of 76% and over 90% in early-stage disease, the number of estimated deaths in 2020 still exceeded 97,000 (1–3). Prognosis of patients with EC depends on pathomorphological as well as molecular characteristics of tumors, the latter becoming an integral part of the latest World Health Organization (WHO) Classification of Female Genital Tumors (2, 4, 5). Currently, four molecular subtypes of EC have been proposed based on genetic characteristics of tumors: (i) POLE (DNA Polymerase Epsilon) ultra-mutated tumors, (ii) mismatch repair-deficient (MMRd) tumors, (iii) p53-mutant tumors (p53abn), and (iv) tumors of no specific molecular profile (NSMP) (5). Molecular classification of EC has offered new insight in the process of carcinogenesis and progression of EC and it has provided new potential targets for treatment and different prognostic subgroups of patients (6–8).

One of important mechanisms that has been linked to tumorigenesis as well as progression of EC is dysfunction of Wnt/β-catenin signaling pathway (9–11). The canonical Wnt/β-catenin signaling pathway is activated by binding of Wnt ligands to heterodimers of Frizzled (FZD) receptors and lipoprotein receptor-related protein (LRP) co-receptors on the surface of the cell. This leads to inactivation of β-catenin destruction complex in the cytoplasm, enabling β-catenin to be transferred to the nucleus, where it forms a complex with the lymphoid enhancer factor (LEF) and T-cell factor (TCF), leading to transcription of cell cycle regulator genes (11, 12). Mutations of catenin beta-1 (*CTNNB1*) gene, occurring in approximately 20-25% of ECs, present an alternative way of activating Wnt/β-catenin signaling pathway through inefficient destruction of β-catenin. Clinically relevant mutations in exon 3 of *CTNNB1* gene prevent phosphorylation and ubiquitination of the protein hence having the same result as binding of Wnt ligands (11, 13–15). Mutations of *CTNNB1* are characteristic for NSMP molecular subtype of EC. Wnt signaling is tightly regulated by Wnt inhibitors, among them the group of Dickkopf (DKK) proteins. Among four members of DKK family proteins, DKK1 – competitive inhibitor against Wnt3a is a prototypical Wnt antagonist and the most extensively studied DKK protein (16). Dysregulation of DKK1 has recently emerged as a potential biomarker of cancer progression and prognosis for several types of malignancies. Its overexpression in endometrial cancer suggests a negative feedback loop between DKK1 expression and Wnt signaling activation (17, 18). Wnt signaling is also regulated by steroid sex hormones. Estrogens and progesterone maintain a dynamic balance between the proliferation and differentiation of endometrium, that is essential for the prevention of abnormal endometrial growth that rises the risk of developing EC (19). Expression of β-catenin was found to be positively correlated with the expression of ER receptors in EC, suggesting a synergy between estrogens and Wnt signaling (10). On the other hand, intact progesterone signaling has a potential to inhibit Wnt signaling by induction of Wnt inhibitors, such as forkhead box 1 protein (FOXO1) (20, 21). Apart from rather well-established role of estrogens and progesterone in the carcinogenesis of EC, androgen receptor (AR) expression also contributes to the progression and prognosis of the disease and may be connected to the Wnt signaling. AR expression in EC is more commonly present in primary tumors and is often lost in metastatic disease (22–24). Tangen et al. discovered a correlation between the loss of AR expression and more aggressive nature of EC and worse prognosis in EC patients (25). Although a correlation between the Wnt signaling and expression of AR has been studied in some cancers (26, 27), there are no studies in EC yet.

Another important mechanism of carcinogenesis and progression of EC is epithelial-to-mesenchymal transition (EMT). EMT leads to the loss of intracellular junctions and of apical-basal polarity in tumor cells as well as the reorganization of the cytoskeleton, increased motility of individual cells, and degradation of the extracellular matrix proteins (6). One of the hallmarks of EMT is the loss of epithelial surface markers, most notably E-cadherin and subsequently the expression of mesenchymal markers, such as N-cadherin and vimentin (28–30). The phenomenon of balanced downregulation of E-cadherin expression and N-cadherin overexpression is described as “cadherin switch” and is regarded as a marker of EMT (6, 30). Wnt signaling is needed for both, the initiation and the maintenance of the mesenchymal phenotype of tumor cells which is mainly achieved by activating the transcription of target genes, contributing to EMT process (31, 32) and is also connected to loss of E-cadherin expression, enabling translocation of β-catenin to participate in Wnt cascade (33). A connection between the Wnt signaling and EMT suggests that Wnt inhibitors, such as DKK proteins could prevent the EMT, making them potential therapeutical targets (18, 20, 34).

This research aims to evaluate the correlation of clinico-pathological and traditional molecular markers of EC with novel biomarkers implicated in more/less aggressive subtypes of EC. Through analyzing the interconnection of Wnt signaling markers and EMT markers the aim of this research is to elucidate the potential role of these novel candidates in prognosis of EC.

## 2 Material and methods

### 2.1 Patient selection and characteristics

This was a prospective cohort study, including all consecutive patients between January 2020 and March 2022 at the University Medical Centre Maribor. All recruited patients underwent surgical treatment of EC after a multidisciplinary tumor board evaluation. Patients’ demographic data such as age at the time of diagnosis, BMI and clinical data were recorded. Exclusion criteria were treatment for benign or pre-cancerous conditions or if there was no available tissue for additional IHC staining.

### 2.2 Molecular analysis

Molecular analysis was performed as previously described using the integrated molecular characterization approach (35). Mutational status of *CTNNB1* gene was determined by Sanger sequencing of exon 3 following the previously described methodology (35). Identification of clinically relevant (15) missense mutations of the following amino acids: D32, G34, S33, S37, T41, D207 and V516, the last one being a splice site variant.

### 2.3 Histopathological characteristics and immunohistochemistry

Standardized pathohistological assessment and additional immunohistochemical staining was performed on post-operative specimens with confirmed endometrial cancer. In 4% of cases (n=3) post-operative specimen showed only benign tissue. In these cases, additional immunohistochemical staining was performed on pre-operative biopsy samples. All samples were assessed at the Department of Pathology of the University Medical Centre Maribor. During routine clinical evaluation, samples are assessed for estrogen (ER), progesterone (PR) expression and morphological characteristics contributing to clinical decision-making as previously described in (35).

In addition to standardized pathology report, samples were selected for ancillary immunohistochemical staining. Paraffine embedded tissue blocks were selected and 4 μm thick slices of tumor tissue were transferred in sections to SuperFrost slides (Thermo Fisher Scientific). Immunostaining was done by standard method in an automatic stainer (BenchMark ULTRA, Ventana Medical Systems, Inc.). Immunostaining was performed for androgen receptors (AR) (rabbit monoclonal antibody, clone SP107, F. Hoffmann-La Roche Ltd., RTU), MMRd (MLH1, MSH2, PMS2, MSH6), p53, β-catenin (mouse monoclonal antibody; clone 17C2; Dako Cytomation Glostrup; at 1:10 dilution), E-cadherin (mouse monoclonal antibody; clone NCH-38; Dako Cytomation Glostrup; at 1:40 dilution), N-cadherin (mouse monoclonal antibody; clone 5D5; GeneTex; at 1:1000 dilution) and DKK1 (rabbit monoclonal antibody; clone SC03-86; Invitrogen – ThermoFisher; at 1:100 dilution). We used marker specific positive and negative controls on every slide. Standard tissue block no. 1 (liver, tonsil, and pancreatic tissue) was used as control for E-cadherin and β-catenin staining. To test and optimize the protocols for N-cadherin and Dkk1, tissue controls were chosen according to the manufacturer’s recommendation – hepatocellular carcinoma, colorectal carcinoma, and placental tissue. Reactions in control tissue are shown in **Supplementary Figure 1**. N-cadherin had overall stronger immuno-positive reactions, compared to Dkk1. A research protocol for N-cadherin and Dkk1 staining was established for EC evaluation. Both markers are not used in routine clinical practice. Since E-cadherin and beta-catenin are validated and frequently used molecular markers in clinical practice, standard tissue blocks were used as controls. Overall reactions in control tissue were similar on every slide with almost no difference in staining intensity. To test and optimize the protocols for N-cadherin and Dkk1, tissue controls were chosen according to the manufacturer’s recommendations. There were internal negative controls in the chosen tissue samples. Reaction of markers with tissue controls is shown in **Supplementary Figure 1**.

**Figure 1:**
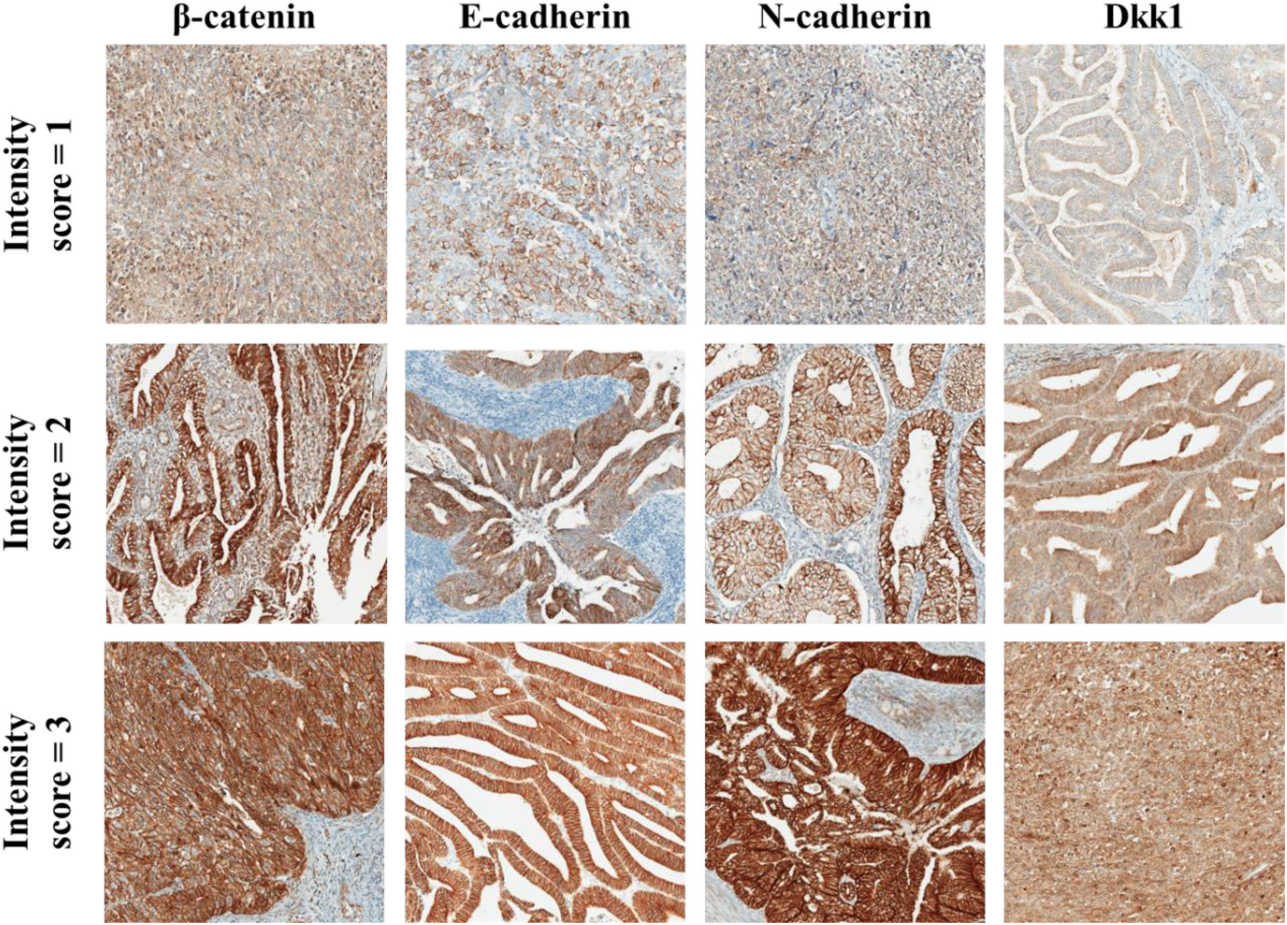
Examples of IHC staining for β-catenin, E-cadherin, N-cadherin, and Dkk1 in EC tumour cells. Staining interpretation was done by assessing staining intensity as weak (intensity score = 1), moderate (intensity score = 2) and strong (intensity score = 3). Intensity score was multiplied by the % of tumour cells with positive reaction and the result was recorded as H score (range from 0 to 300). All micrographs are taken at 100x magnification.

The sample evaluation was done independently by two pathology experts (DS and MH), who were blinded to each other’s grades. If there was discrepancy between their grading, higher than 20%, samples were re-evaluated, and the experts settled a final score. Expression of all immunohistochemical markers (β-catenin, Dkk1, E-cadherin and N-cadherin) was evaluated by counting the number of immuno-positive tumor cells and expressed by percentage. Tumor cells were deemed positive if there was a clear membranous (β-catenin) (36, 37), membranous and/or cytoplasmic (E-cadherin, N-cadherin) (29, 38) and cytoplasmic (Dkk1) (10, 39, 40) reaction. At the same time, intensity of reaction was scored on the scale from 0 to 3 (0 = no reaction, 1 = mild, 2 = moderate, 3 = strong). Examples of scoring system is shown in **Figure 1**. Using the described parameters, we calculated the standard H score, as previously reported (39, 41). H score was calculated by multiplying the percentage of positive cells with staining intensity. Additionally, we recorded the β-catenin nuclear staining, that could be used as a surrogate for Wnt signaling, in tumor cells and categorized it as either focal or diffuse, based on the extent of nuclear reaction in tumor cells (42, 43).

Whole Slide Images (WSI) were taken using Aperio ScanScope CS under the same conditions. WSI were then exported as.jpeg format using Aperio Slide Manager software and were not edited, only cropped to the same size.

### 2.4 Statistical analysis

Descriptive analysis was used for numeric variables, using median (Me) and range. Absolute and relative frequencies were reported for categorical variables. Expression levels of molecular markers were all expressed as median value or either % of expression or median H-score. To assess the correlation between two numeric variables, namely the % of hormone receptor expression and H-score of other molecular marker expression, Spearman correlation coefficient was calculated, and scatter plot diagrams (**Supplementary Material**) were used to present the results. Correlations between numeric and categorical variables were evaluated using non-parametric tests, either Mann-Whitney U test or Kruskal-Wallis H-test. Results were presented by reporting U value when Mann-Whitney U test was used and H value when Kruskal-Wallis H test was used, along with the level of significance (p value). Statistical significance was set at *p*<0.05. Statistical analysis was performed using SPSS for Windows, Version 25.0.0 (IBM Corp., Armonk, NY, USA).

## 3 Results

### 3.1 Patient characteristics

Sixty-five women were included in this study. Their clinico-pathological characteristics are depicted in **Table 1**.

**Table 1:** Patient characteristics.

**Median age at time of diagnosis (n=65)**			69 years (41-87)	
**Median Body Mass Index (BMI) (n=65)**			31 17-43)	
			n (%)	CI 95%
**Menopausal status**	Pre-menopausal		6 (9%)	[4-10]
	Post-menopausal		59 (91%)	[82 - 96]
**EC subtype**	Type I		55 (85%)	[74 - 92]
	Type II		10 (15%)	[8 - 26]
**EC grade**	Low grade (G1-2)		50 (88%)	[77 - 94]
	High grade (G3)		7 (12%)	[6 - 23]
**LVSI**	absent		46 (71%)	[59 - 81]
	focal		2 (3%)	[1 - 10]
	diffuse		17 (26%)	[17 - 38]
**Myometrial invasion**	≤ 50%		32 (49%)	[37 - 61]
	> 50%		33 (51%)	[39 - 63]
**FIGO stage**	Stage I	IA	32 (49%)	[37 - 61]
		IB	16 (25%)	[15 - 36]
	Stage II		1 (2%)	[0 - 7]
	Stage III		13 (20%)	[12 -30]
	Stage IV		3 (5%)	[1 - 12]
**Integrated molecular subgroup**	POLEmut		4 (6%)	[2 - 14]
	MMRd		23 (35%)	[25 - 47]
	NSMP among them CTNNB1mut		32 (49%) 4 (6%)	[37 - 61]
	p53abn		6 (9%)	[4 - 18]
**ESGO-ESTRO-ESP patient risk assessment**	low risk		29 (45%)	[33 - 57]
	intermediate risk		8 (12%)	[6 - 22]
	high-intermediate risk		4 (6%)	[2 - 14]
	high risk		21 (32%)	[21 - 44]
	advanced carcinoma		3 (5%)	[1 - 12]

EC, Endometrial cancer; LVSI, Lympho-vascular infiltration; FIGO stage, The International Federation of Gynecology and Obstetrics; ESGO-ESTRO-ESP, European Society for Gynaecologic Oncology - European Society Radiation Oncology – European Society for Pathology.

### 3.2 Correlation between expression of molecular markers

**Figure 2:**
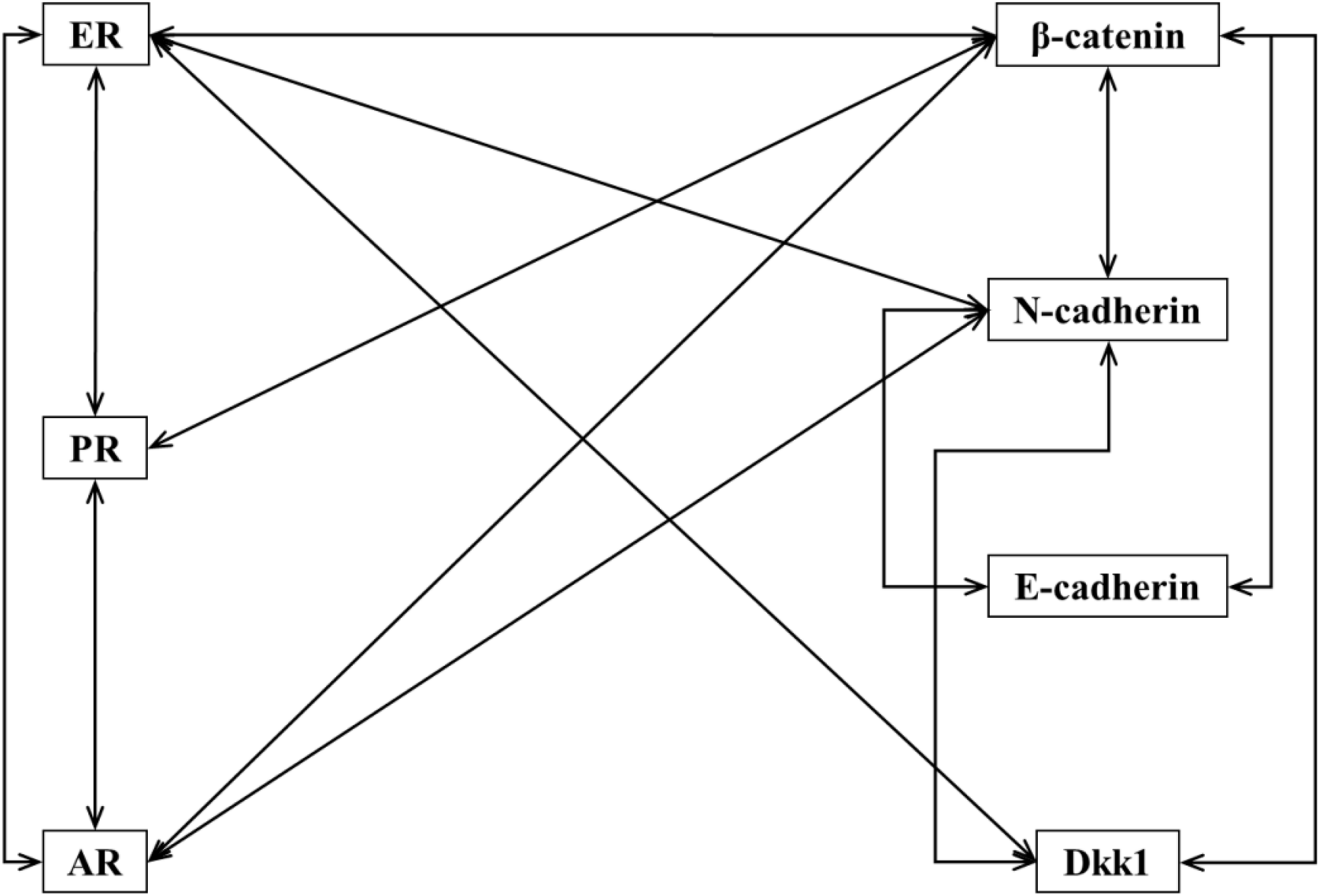
Correlations between expression of different molecular markers. Lines connect IHC markers with positive and statistically significant correlation in their expression in tumours cells.

Expression of selected molecular markers (β-catenin, E-cadherin, N-cadherin and Dkk1) and hormone receptors (ER, PR and AR) in tumour tissue was evaluated and is presented in **Figure 2** and **Supplementary Table 1**. Expression of ER was found to be positively correlated with expression of PR (r(64) = 0.844, p<0.05), AR (r(55) = 0.597, p<0.05), β-catenin (r(64) = 0.065, p<0.05), N-cadherin (r(64) = 0.280, p<0.05) and Dkk1 (r(64) = 0.263, p<0.05). Expression of PR was positively correlated with expression of ER, AR (r(55) = 0.554, p<0.05) and β-catenin (r(65) = 0.287, p<0.05). Expression of AR was positively correlated with expression of ER, PR, β-catenin (r(55) = 0,308, p<0.05) and N-cadherin (r(55) = 0.332, p<0.05). Expression of β-catenin was positively correlated with expression of ER, PR, AR, E-cadherin (r(65) = 0.345, p<0.05), N-cadherin (r(65) = 0.649, p<0.05) and Dkk1 (r(65) = 0.392, p<0.05). Expression of E-cadherin was positively correlated with expression of β-catenin and N-cadherin (r(65) = 0.452, p<0.05). Expression of N-cadherin was positively correlated with expression of ER, AR, β-catenin, E-cadherin and Dkk1 (r(65) = 0.365, p<0.05). Expression of Dkk1 was positively correlated with expression of ER, β-catenin and N-cadherin.

### 3.3 Expression correlation of β-catenin with EMT markers and hormone receptors in EC

Nuclear expression of β-catenin was found in 45 (69.2%; 95% CI [57.4%, 79.4%]) ECs. Pattern of expression was mostly focal with smaller groups of tumour cells showing positive nuclear reaction. Diffuse nuclear positivity was shown in 2 cases (3,1%).

We compared the membranous expression of β-catenin, cadherins and Wnt antagonist Dkk1 in tumour tissue as well as expression of hormone receptors (ER, PR and AR) against nuclear expression of β-catenin. Results are shown in **Table 2**. Expression of N-cadherin was higher for tumours with nuclear expression of β-catenin (Me [H-score] = 270) than for tumours without nuclear β-catenin expression (Me [H-score] = 253), U = 296.5, p < 0.05. Expression of Dkk1 was also found to be higher for tumours with nuclear expression of β-catenin (Me [H-score] = 115) than for tumours without nuclear β-catenin expression (Me [H-score] = 102), U = 270.5, p < 0.05. Expression of membranous β-catenin was found to be higher in tumours with nuclear β-catenin expression (Me [H-score] = 250), compared to tumours without nuclear β-catenin expression (Me [H-score] = 235), U = 254.5, p < 0.05. There was no statistically significant correlation between nuclear expression of β-catenin and the expression of E-cadherin. Expression of ER receptors was higher in tumours with nuclear expression of β-catenin (Me [%] = 100) compared to tumours without nuclear expression of β-catenin (Me [%] = 70), U = 183.0, p < 0.05. The same was true for the expression of PR in tumours with nuclear expression of β-catenin (Me [%] = 100) versus without (Me [%] = 45), U = 180.0, p < 0.05 and for expression of AR in tumours with nuclear expression of β-catenin (Me [%] = 30) versus without (Me [%] = 10), U = 163.0, p < 0.05.

**Table 2:** Correlations between expression of hormone receptors and molecular markers in tumours with and without nuclear expression of β-catenin.

Molecular marker	Nuclear expression of β-catenin		
	PRESENT (Median value)	ABSENT (Median value)	Mann-Whitney U-test
ER	100%	70%	U = 183.0; p < 0.05
PR	100%	45%	U = 180.0; p < 0.05
AR	30%	10%	U = 163.0; p < 0.05
β-catenin (membranous)	H score: 250	H score: 235	U = 254.5; p < 0.05
E-cadherin	H score: 223	H score: 223	U = 430.5; p = 0.782
N-cadherin	H score: 270	H score: 253	U = 296.5; p < 0.05
Dkk1	H score: 115	H score: 102	U = 270.5; p < 0.05

### 3.4 Correlation between expression of molecular markers and clinical characteristics

Expression of molecular markers (β-catenin, E-cadherin, N-cadherin and Dkk1) and hormone receptors (ER, PR and AR) was compared to clinical data and histopathological characteristics of the tumours, listed in **Table 1**. All results are shown in **Supplementary Tables 2**, **3**.

Comparison between the ESGO-ESTRO-ESP patient risk assessment categories and expression of molecular markers showed significant correlations with the expression of Dkk1, H = 10.196, p < 0.05. The expression of Dkk1 was lower in low-risk (H-score = 105) and high-intermediate risk groups (H-score = 65), compared to intermediate-risk (H-score = 112) ang high-risk (H-score = 111) groups, but was highest in advanced carcinoma (H-score = 130). There was no significant change between expression of hormone receptors or other markers and patient risk groups.

There was no significant difference between expression of any of the molecular markers or hormone receptors and presence of LVSI, stage of the disease, myometrial invasion, FIGO stage or integrated molecular subgroups as shown in **Supplementary Table 2**. Higher expression of ER and PR was detected in Type I compared to Type II tumour and in low grade compared to high grade tumours.

## 4 Discussion

This prospective study shows that Wnt signalling is significantly involved in driving behaviour of endometrial cancer. Wnt signalling and expression of EMT markers in EC were significantly correlated with hormone receptors status in EC, but not with other clinico-pathological characteristics. Expression of Wnt antagonist, Dkk1 was significantly different among the ESGO-ESTRO-ESP patient risk assessment categories, being the highest in advanced carcinoma, lowest in high-intermediate risk group and approximately the same in low-risk, intermediate-risk, and high-risk groups.

The historical discrimination between type I and type II EC shows the influence of hormone status on pathogenesis and progression of EC. Recent studies have elucidated the impact of ER and PR expression on clinicopathological characteristics of EC, such as tumour invasiveness and FIGO stage. Loss of hormone receptor expression has been linked to worse prognosis and lower overall survival of patients with EC (44–47). Results of our study showed significant difference between the ER and PR expression, tumour type, and tumour grade, but not other clinicopathological characteristics. Most likely explanation why our results do not concur with previous studies is that our study compared a combined H-score with other tumour characteristics, whereas most of other studies used two-tier grading of hormone receptor status (positive or negative) and only set a specific cut-off value. Our approach has also been suggested to be more appropriate in clinical practise (48).

Important mechanism of EC carcinogenesis is Wnt signalling pathway. Its result is translocation of β-catenin into the cell nucleus, triggering target gene expression of cell cycle regulators. Nuclear β-catenin expression, determined by IHC, has been widely studied as a potential surrogate for Wnt signalling and *CTNNB1* gene mutations. Such mutations of exon 3 in *CTNNB1* gene occur in up to 20% of tumours, more often in low grade, early ECs (15). In our study 6% of women with EC had *CTNNB1* mutations, which is lower than expected (49, 50). *CTNNB1* mutational status in EC was not associated with any clinicopathological characteristics of the tumours, or with expression of hormone nor other molecular markers. However, it is possible to assess presence of Wnt signalling by IHC determination of β-catenin, regardless of mutational status of *CTNNB1* gene (36, 43).

Comparing Wnt signalling to expression of other markers in this study revealed positive correlation with the expression of all hormone receptors, membranous expression of β-catenin and expression of N-cadherin as well as Wnt antagonist, Dkk1. Wnt signalling in normal endometrium is regulated also by the expression of Wnt antagonists, such as Dkk1. Our results showed higher expression of Dkk1 in tumours with nuclear β-catenin expression, suggesting negative feedback loop between Wnt signalling and Wnt antagonists, as has been proposed by previous studies (18, 51). So far Dkk1 expression was found to be higher in benign endometrial tissue, compared to EC and was also found to be higher in low grade EC compared to high grade EC (10, 40), supporting the theory of downregulation of Wnt antagonists’ expression in EC (17, 52). Our results were in concordance with research done so far (10, 40), we showed lower expression of Dkk1 in high grade EC compared to low grade EC, but the difference was not statistically significant. We are among the first to compare expression of Dkk1 to molecular characteristics of EC, as well as integrated risk groups, based on new ESGO-ESTRO-ESP guidelines. Studies that have compared IHC expression of Dkk1 among different FIGO stages or histological grades of EC so far are scarce (10, 40, 53) and do not consider the potential influence of molecular classification. We did not find any significant correlation between molecular groups themselves, but we found the expression of Dkk1 to be significantly different between ESGO-ESTRO-ESP risk groups, being upregulated in advanced carcinoma. However, our results did not show linear increase in Dkk1 expression across the integrated risk groups, which could be a consequence of a small sample size. ESGO-ESTRO-ESP risk groups are based on a combination of pathohistological characteristics (histological type, tumour grade, LVSI), FIGO grade and molecular classification of EC (4). Since Dkk1 could be one of potential therapeutic targets (52, 54) further studies are needed to determine, whether there is a difference between expression of Dkk1 in tumour tissue and serum of the patient and how any of those would influence the potential use of therapeutics for EC.

Alterations in cellular adhesion molecules, are important mechanism of tumour progression and metastasis. Lower expression of membranous E-cadherin or complete loss of E-cadherin expression has been associated with higher FIGO grade, deep myometrial invasion, risk of tumour recurrence, and metastatic disease (36, 55, 56). Our study showed similar patterns of lower E-cadherin expression in tumours of Type II compared to Type I endometrial cancer, presence of LVSI and deeper myometrial invasion, but not in tumours of higher grade or higher FIGO stage. However, none of our results were statistically significant. Other authors have reported correlation between low expression of E-cadherin and higher expression of other cadherins, most importantly N-cadherin, marker of mesothelial differentiation and thus indicator of EMT (6, 38, 57). Our study, on the contrary, showed a positive correlation between expression of both cadherins, a phenomenon that has not yet been recognized. The phenomenon, called “cadherin switch” has been implicated, often described in other types of cancer, i.e. breast cancer or ovarian cancer (58, 59), but also in EC (29). We compared the expression of cadherins with nonparametric test, comparing the mean H-score value, like other studies (39, 41), since cut-off for defining positive or negative expression of cadherins has not been validated in any of the previous studies. In comparison to most other studies, we studied the average overall expression of cadherins in tumour tissue. We did not compare or distinguish between only membranous or cytoplasmic staining to take into an account a possible different intracellular location of the marker, also we did not compare the IHC reaction in centre of the tumour-to-tumour front, where differences have been most observed (6, 38). Our N-cadherin staining has been very strong overall, having a very high mean H-score, regardless of tumour type, stage, or grade.

There are different limitations of this study which are connected to the explorative nature of the methodology as well as the cohort itself. As previously discussed in cancers, where IHC receptor expression is important in therapeutic decision-making, cut-off values for predictive outcomes need to be validated in larger cohorts. While there has been advancement in our understanding of appropriate hormone receptor (ER, PR) cut-offs in EC, no such cut-offs are determined for EMT markers and Wnt markers in EC. IHC methods for assessment of molecular markers need broader criteria validation for assessment of EMT levels and Wnt marker cut-off values. Our explorative study has added to this understanding, but further evaluation is needed to test against specific cut-off values in subgroups of EC. Validation of potential biomarkers is an extensive process evaluating the rationale, mechanism and impact a certain molecule has on the process of carcinogenesis. Several recommendations suggest the use of archival samples and prospective samples as the first steps in the biomarker discovery process (60). These need to be followed or developed in parallel by translational validation using Western blot validation. This enables further protein identification and quantification and thus better understanding of the mechanism of action (61). Due to the limited resources, Western blot has not been performed in our study yet. Furthermore, in improving our mechanistic understanding of the topic, IHC is only the first step in Wnt signalling evaluation. Since we had very small group of tumours with *CTNNB1* mutations, we could not study the effects of alternative activation of Wnt pathway and its potential influence on EMT. Further studies are needed to address the different activation mechanisms of Wnt pathway and its connection to Wnt antagonists to evaluate the possible effects of guiding therapy for EC. Lastly, the results, due to its pilot nature, need to be cautiously evaluated due to a small number of cases reported. This cohort provides insight into the topic, yet larger subgroup analyses are needed to show utility for further translational understanding.

## 5 Conclusions

Our data indicates that Wnt signalling (nuclear β-catenin expression) in EC could be correlated to markers of EMT (N-cadherin), Wnt antagonist (Dkk1) and hormone receptors. Although this should be verified on larger population of EC patients, our data provides new insight into signalling pathways in EC. Correlation between expression of hormone receptors and other molecular markers affirms the connection between Wnt and EMT pathways in EC. Significant difference between expression of Dkk1 among ESGO-ESTRO-ESP patient risk assessment categories contributes to a better understanding of its role in EC with further implications for research of potential target immunotherapy.

## Data availability statement

The raw data supporting the conclusions of this article will be made available by the authors, without undue reservation.

## Author contributions

Conceptualization: ŽL, MS, and JK. Methodology: ŽL and MS. Formal analysis: DS, MH, TB, MS, ŽL, and JK. Resources: JK, MS, and UP. Data curation: DS, MH, TB, MS, ŽL, and JK. Writing—original draft preparation: ŽL and MS. Writing—review and editing: ŽL, MS, and JK. Visualization: ŽL. Supervision: JK and UP. Project administration: MS. Funding acquisition: JK. All authors contributed to the article and approved the submitted version.
