# Supplementary material for: The association of Wnt-signalling and EMT markers with clinical characteristics in women with endometrial cancer
Source: Front Oncol. 2023 Mar 8;13:1013463. doi: 10.3389/fonc.2023.1013463 (PMC10031053; doi:10.3389/fonc.2023.1013463)
Supplement: Supplementary file 1 [file DataSheet_1.docx]

**Supplemental data**

Suppl. Table 1: Correlation between expression of molecular marker in EC

| Markers | PR | AR | β-catenin | E-cadherin | N-cadherin | Dkk1 |
| --- | --- | --- | --- | --- | --- | --- |
| ER | r (64) = 0.844 p < 0.05 | r (55) = 0.597 p < 0.05 | r (64) = 0.305  p < 0.05 | r (64) = 0.065  p = 0.610 | r (64) = 0.280  p < 0.05 | r (64) = 0.263  p < 0.05 |
| PR | / | r (55) = 0.554 p < 0.05 | r (65) = 0.287  p < 0.05 | r (65) = 0.123 p = 0.330 | r (65) = 0.221  p = 0.076 | r (65) = 0.213  p = 0.089 |
| AR | r (55) = 0.554. p < 0.05 | / | r (55) = 0.308 p < 0.05 | r (55) = -0.013 p = 0.992 | r (55) = 0.332  p < 0.05 | r (55) = 0.139  p = 0.311 |
| β-catenin | r (65) = 0.287  p < 0.05 | r (55) = 0.308 p < 0.05 | / | r (65) = 0.345  p < 0.05 | r (65) = 0.649  p < 0.05 | r (65) = 0.392  p < 0.05 |
| E-cadherin | r (65) = 0.123 p = 0.330 | r (55) = -0.013 p = 0.992 | r (65) = 0.345  p < 0.05 | / | r (65) = 0.452  p < 0.05 | r (65) = 0.186 p = 0.139 |
| N-cadherin | r (65) = 0.221  p = 0.076 | r (55) = 0.332  p < 0.05 | r (65) = 0.649  p < 0.05 | r (65) = 0.452  p < 0.05 | / | r (65) = 0.365  p < 0.05 |

Suppl. Graph 1-21: Graphical representation of correlation between hormone receptor and other molecular marker expression (green square indicating cases with significant correlation)


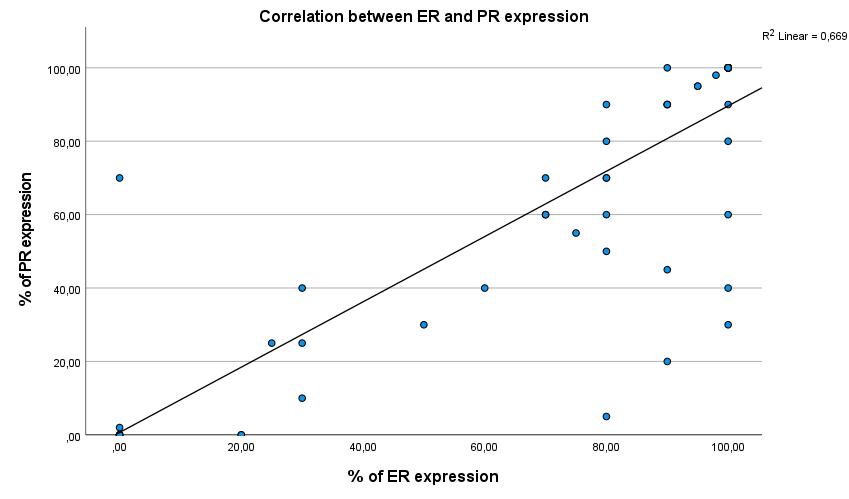

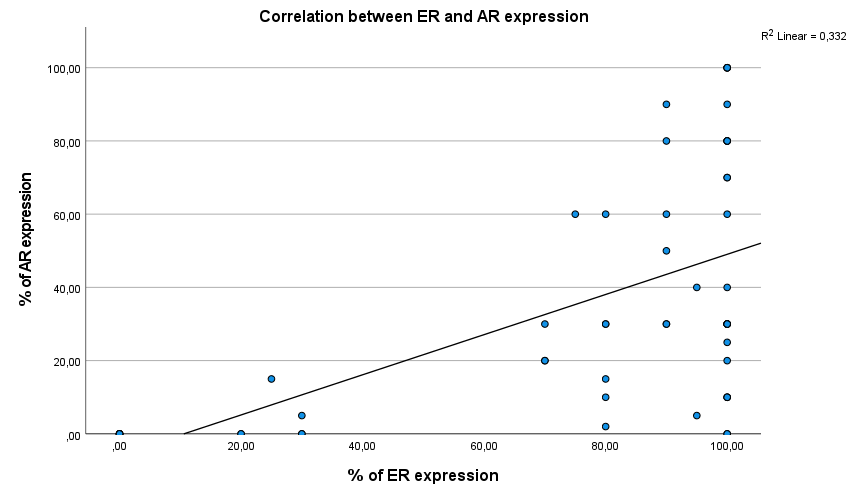


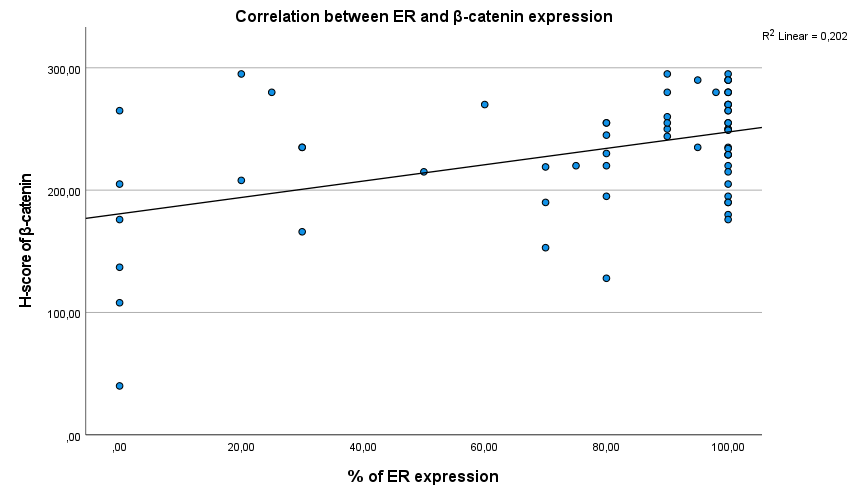

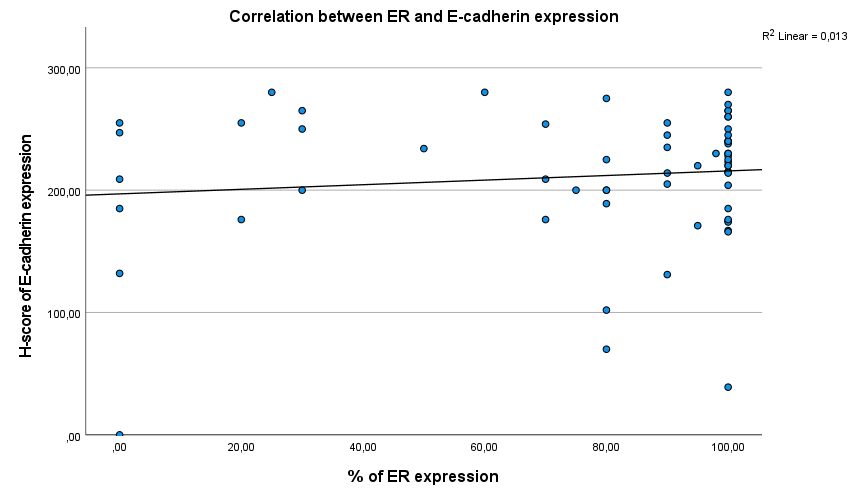


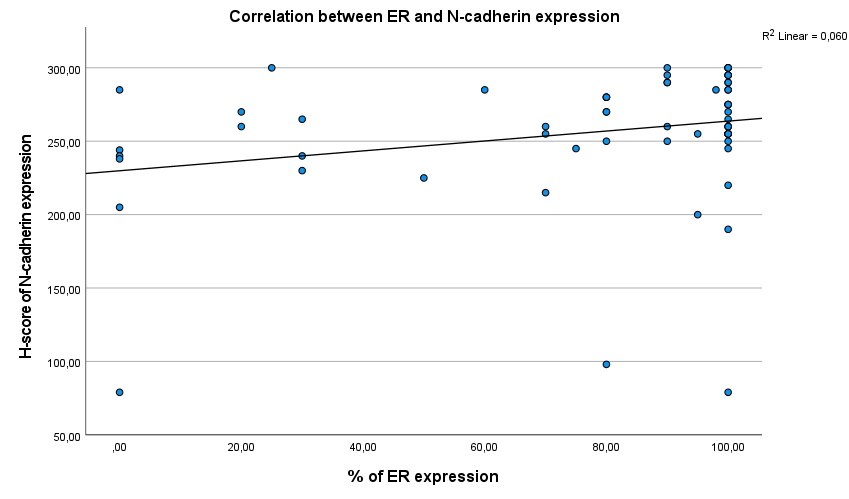

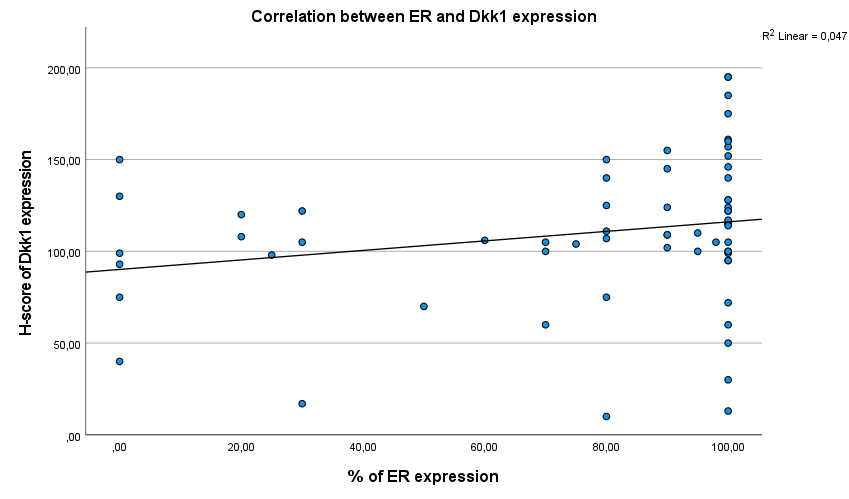


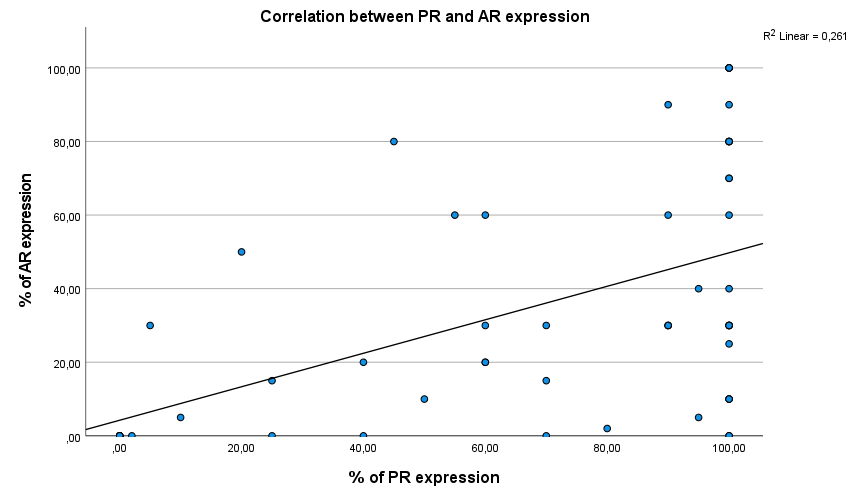

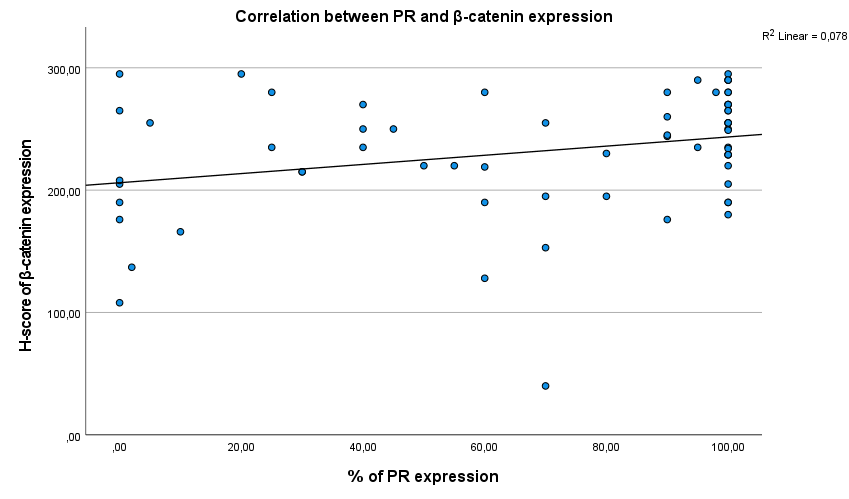


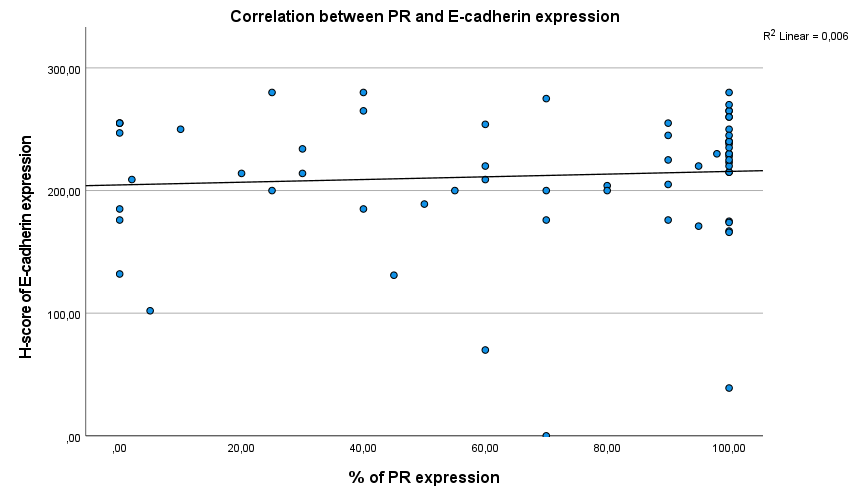

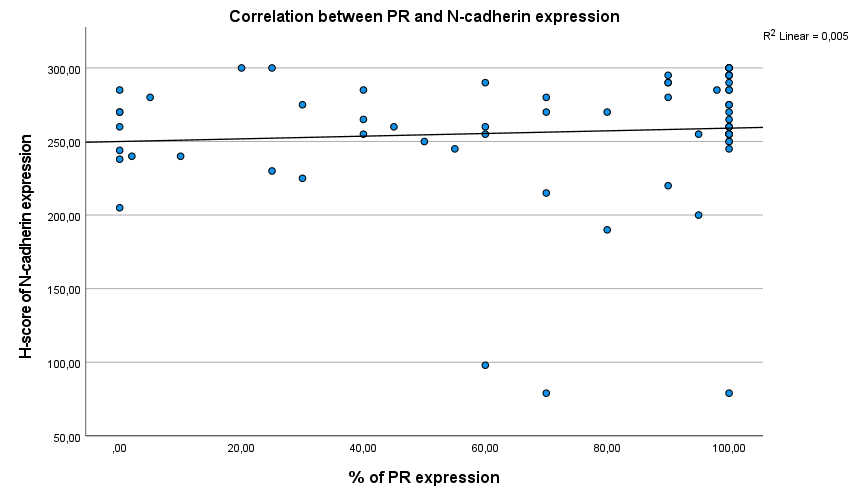


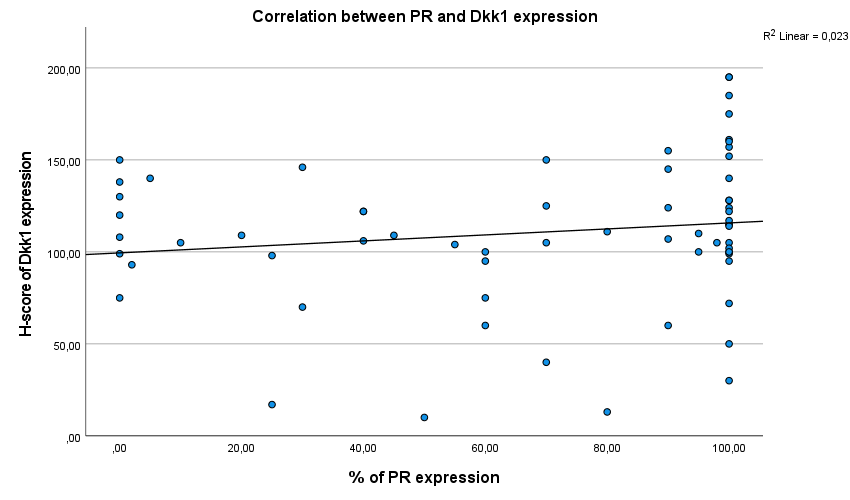

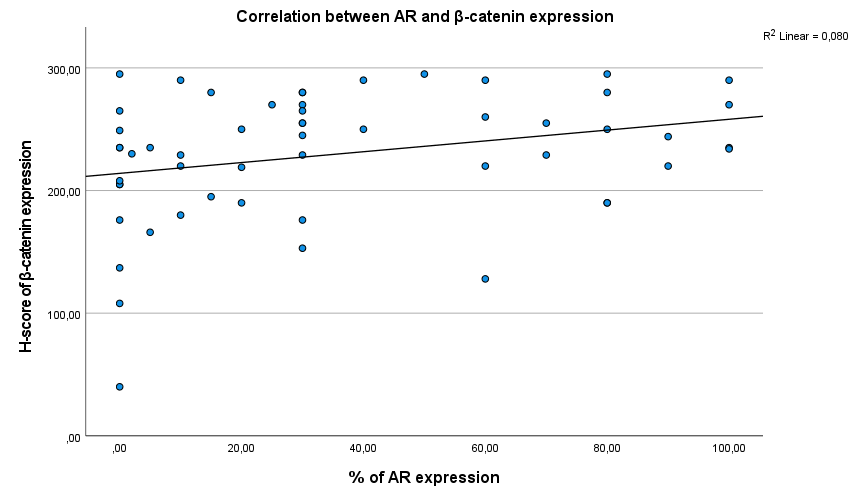


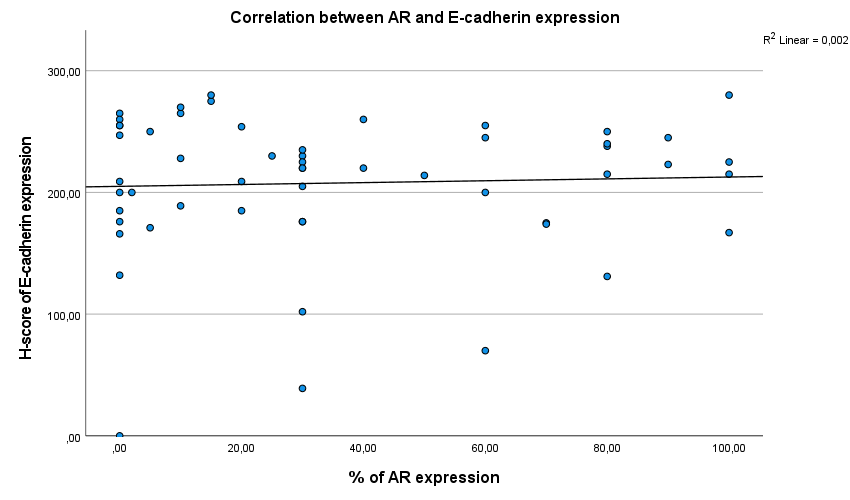

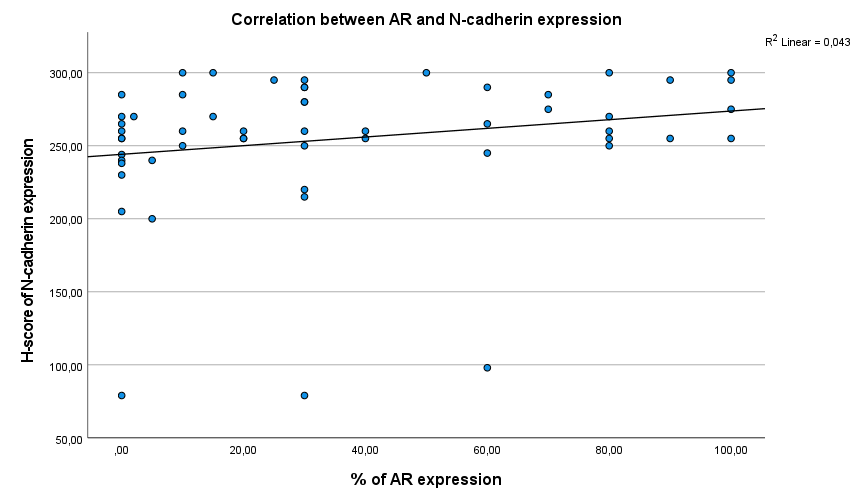


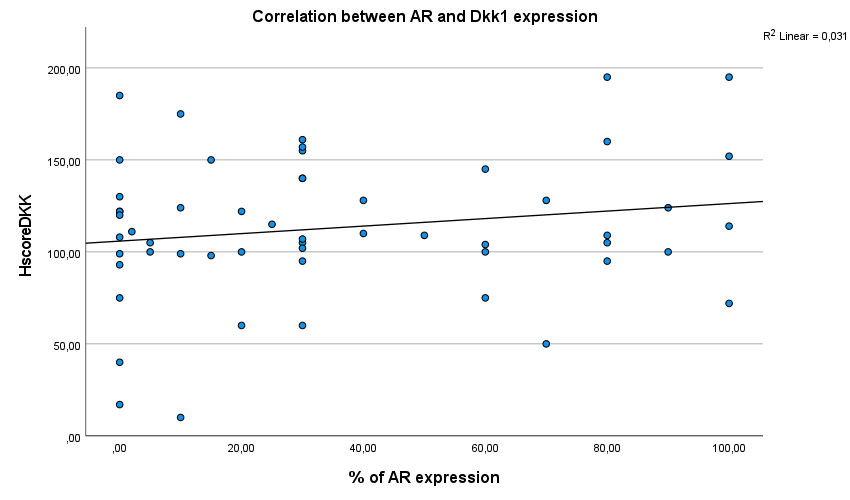

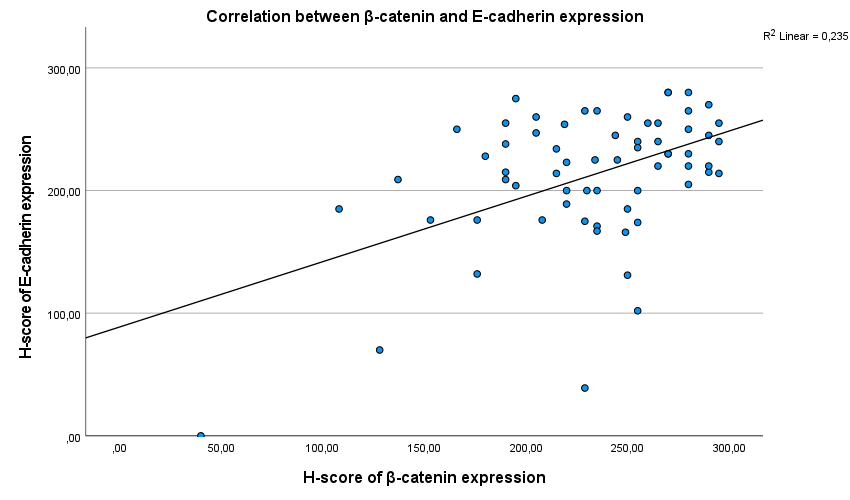


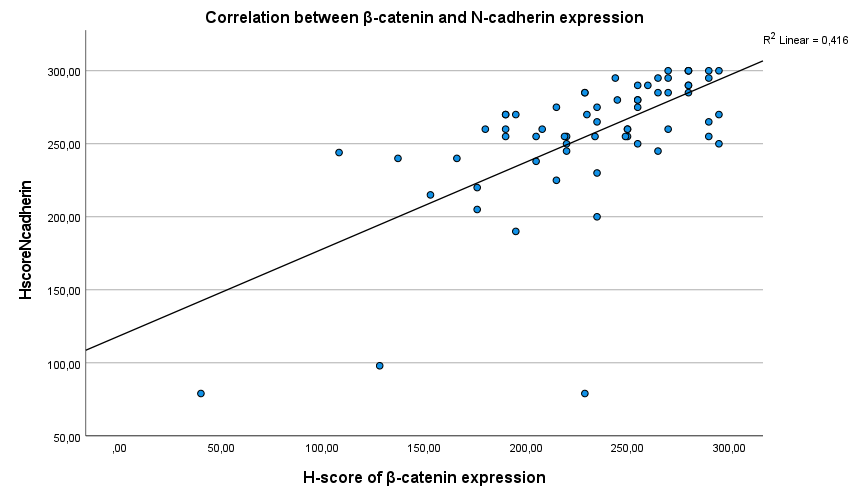

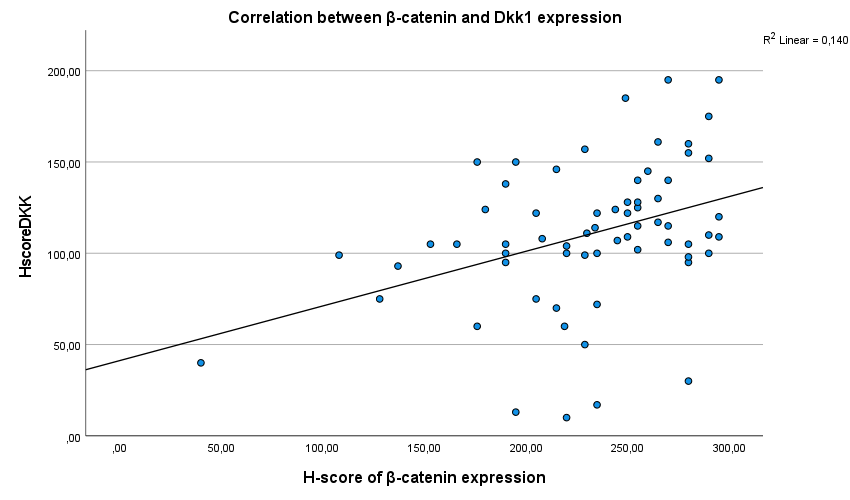


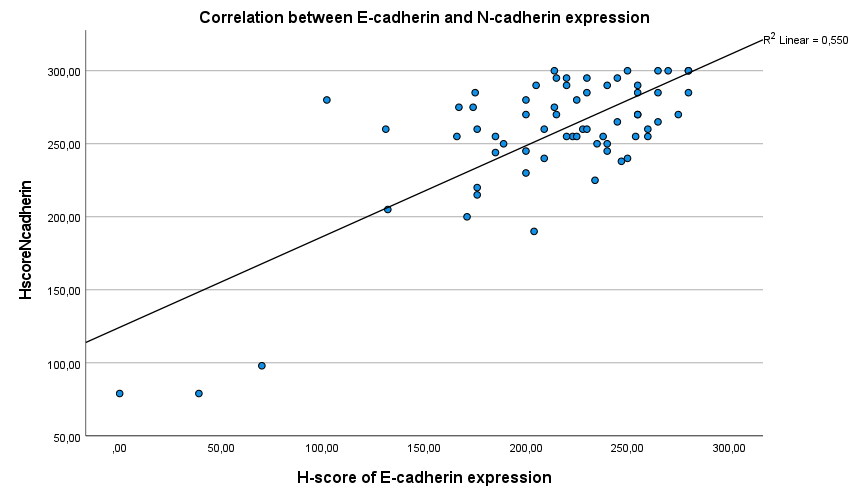

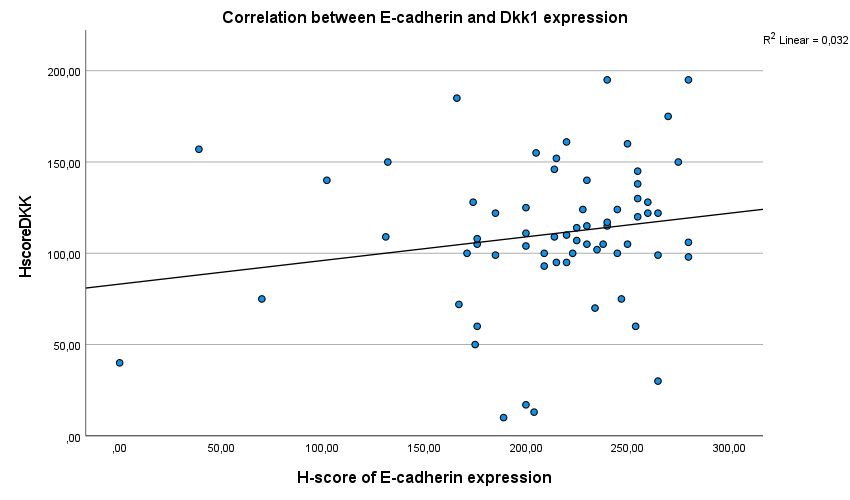


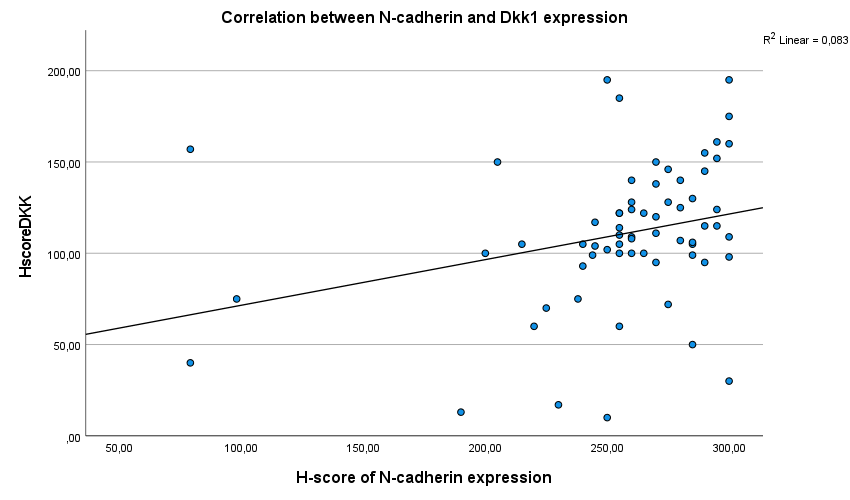


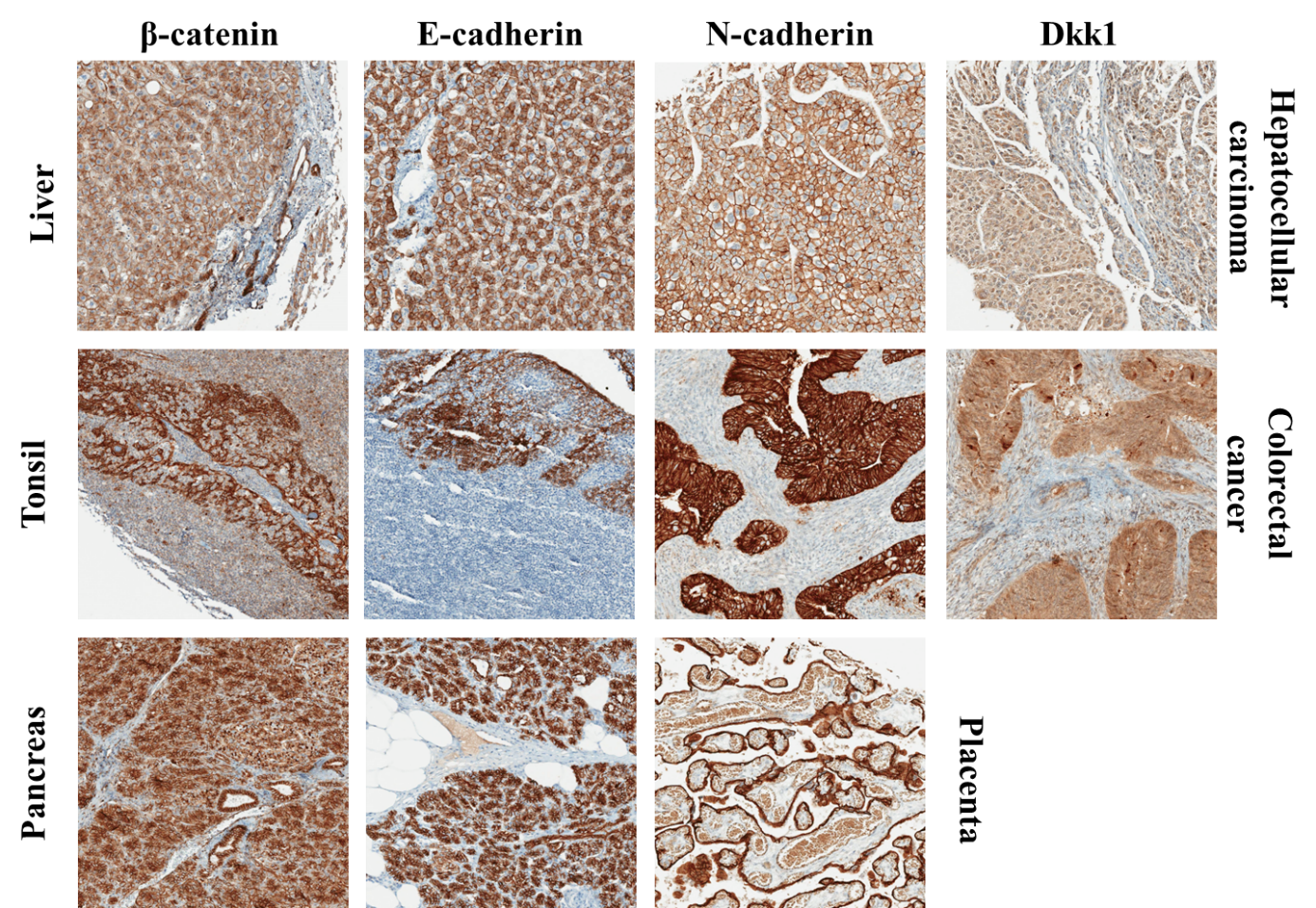


Suppl. Figure 1: Controls used for immunohistochemical analysis of tissue samples.

|  | | **Expression of ER** | | **Expression of PR** | | **Expression of AR** | |
| --- | --- | --- | --- | --- | --- | --- | --- |
| Tumour characteristic | | Median % | Correlation | Median % | Correlation | Median % | Correlation |
| EC subtype | Type I | 100 | U = 104.0  p < 0.05 | 90 | U = 116.5  p < 0.05 | 30 | U = 98.0  p < 0.05 |
|  | Type II | 20 |  | 3 |  | 0 |  |
| EC grade | Low grade | 100 | U = 69.5 p < 0.05 | 95 | U = 69.0 p < 0.05 | 30 | U = 93.0 p = 0.622 |
|  | High Grade | 60 |  | 30 |  | 20 |  |
| LVSI | Absent | 90 | U = 398.0  p = 0.647 | 90 | U = 434.5  p = 0.970 | 30 | U = 310.0  p = 0.677 |
|  | Present | 100 |  | 80 |  | 30 |  |
| Stage of the disease | Early | 93 | U = 344.5  p 0.689 | 90 | U = 371.0  p = 0.935 | 30 | U = 228.0  p = 0.876 |
|  | Advanced | 100 |  | 85 |  | 28 |  |
| Myometrial invasion | ≤ 50% | 100 | U = 443.5  p = 0.334 | 96 | U = 471.5  p = 0.443 | 30 | U = 375.0  p = 1.000 |
|  | > 50% | 90 |  | 70 |  | 30 |  |
| FIGO stage | Stage IA | 98 | H = 1.075  p = 0.898 | 92 | H = 0.555  p = 0.968 | 30 | H = 1.072  p = 0.899 |
|  | Stage IB | 85 |  | 80 |  | 30 |  |
|  | Stage II | 80 |  | 60 |  | 60 |  |
|  | Stage III | 100 |  | 80 |  | 25 |  |
|  | Stage IV | 100 |  | 100 |  | 70 |  |
| Integrated molecular subgroup | POLEmut | 45 | H = 4.758  p = 0.190 | 58 | H = 6.659  p = 0.084 | 20 | H = 0.437  p = 0.932 |
|  | MMRd | 90 |  | 90 |  | 30 |  |
|  | NSMP | 100 |  | 95 |  | 30 |  |
|  | p53abn | 70 |  | 10 |  | 20 |  |
| ESGO-ESTRO-ESP patient risk assessment | Low | 100 | H = 1.257  p = 0.869 | 100 | H = 2.888  p = 0.577 | 30 | H = 4.613  p = 0.329 |
|  | Intermediate | 90 |  | 80 |  | 50 |  |
|  | High-intermediate | 85 |  | 75 |  | 20 |  |
|  | High | 85 |  | 70 |  | 25 |  |
|  | Advanced EC | 100 |  | 100 |  | 70 |  |
| H = H-value of Kruskall-Wallis test used for calculating statistical significance; U = U-value of Mann-Whitney test used for calculating statistical significance | | | | | | | |

Suppl. Table 2: Correlation of hormone receptors (ER, PR and AR) and characteristics of tumours

Suppl. Table 3: Correlation of molecular markers expression and characteristics of tumours

|  | | **Expression of  β-catenin** | | | **Expression of  E-cadherin** | | **Expression of  N-cadherin** | | **Expression of  Dkk1** | |
| --- | --- | --- | --- | --- | --- | --- | --- | --- | --- | --- |
| Tumour characteristic | | Median  H-score | Correlation | Median  H-score | | Correlation | Median  H-score | Correlation | Median  H-score | Correlation |
| EC subtype | Type I | 245 | U = 167.5  p = 0.050 | 225 | | U = 179.5  p = 0.073 | 265 | U = 187.5  p = 0.111 | 110 | U = 275.5  p = 0.750 |
|  | Type II | 207 |  | 181 | |  | 252 |  | 107 |  |
| EC grade | Low grade | 247 | U = 112.5  p = 0.128 | 224 | | U = 150.5  p = 0.551 | 268 | U = 138.0  p = 0.367 | 111 | U = 165.5 p = 0.817 |
|  | High Grade | 215 |  | 234 | |  | 260 |  | 109 |  |
| LVSI | Absent | 245 | U = 378.0  p = 0.394 | 228 | | U = 324.5  p = 0.104 | 265 | U = 386.0  p = 0.461 | 108 | U = 353.0  p = 0.226 |
|  | Present | 229 |  | 200 | |  | 260 |  | 111 |  |
| Stage of the disease | Early | 235 | U = 329.5  p = 0.462 | 225 | | U = 330.0  p = 0.467 | 260 | U = 331.5  p = 0.481 | 106 | U = 265.0  p = 0.080 |
|  | Advanced | 250 |  | 223 | |  | 265 |  | 119 |  |
| Myometrial invasion | ≤ 50% | 242 | U = 483.0  p = 0.237 | 227 | | U = 483.5  p = 0.559 | 263 | U = 518.0  p = 0.895 | 106 | U = 490.5  p = 0.623 |
|  | > 50% | 234 |  | 220 | |  | 260 |  | 111 |  |
| FIGO stage | Stage IA | 235 | H = 6.521  p = 0.163 | 227 | | H = 3.873  p = 0.423 | 265 | H = 3.859  p = 0.425 | 105 | H = 5.914  p = 0.206 |
|  | Stage IB | 229 |  | 223 | |  | 260 |  | 112 |  |
|  | Stage II | 128 |  | 70 | |  | 98 |  | 75 |  |
|  | Stage III | 249 |  | 220 | |  | 260 |  | 111 |  |
|  | Stage IV | 265 |  | 240 | |  | 275 |  | 130 |  |
| Integrated molecular subgroup | POLEmut | 213 | H = 5.588  p = 0.133 | 132 | | H = 5.615  p = 0.132 | 233 | H = 7.522  p = 0.057 | 119 | H = 4.286  p = 0.232 |
|  | MMRd | 229 |  | 215 | |  | 255 |  | 102 |  |
|  | NSMP | 255 |  | 228 | |  | 273 |  | 115 |  |
|  | p53abn | 239 |  | 235 | |  | 270 |  | 115 |  |
| ESGO-ESTRO-ESP patient risk assessment | Low | 235 | H = 5.733  p = 0.220 | 230 | | H = 4.675  p = 0.322 | 260 | H = 2.511  p = 0.643 | 105 | H = 10.196  p < 0.05 |
|  | Intermediate | 239 |  | 218 | |  | 270 |  | 112 |  |
|  | High-intermediate | 217 |  | 244 | |  | 240 |  | 65 |  |
|  | High | 245 |  | 200 | |  | 260 |  | 111 |  |
|  | Advanced EC | 265 |  | 240 | |  | 275 |  | 130 |  |
| H = H-value of Kruskall-Wallis test used for calculating statistical significance; U = U-value of Mann-Whitney test used for calculating statistical significance | | | | | | | | | | |
